# Genomic prediction of avian influenza infection outcome in layer chickens

**DOI:** 10.1186/s12711-018-0393-y

**Published:** 2018-05-02

**Authors:** Anna Wolc, Wioleta Drobik-Czwarno, Janet E. Fulton, Jesus Arango, Tomasz Jankowski, Jack C. M. Dekkers

**Affiliations:** 1 Department of Animal Science, Iowa State University, 806 Stange Road, 239E Kildee Hall, Ames, IA 50010 USA; 2 Hy-Line International, 2583 240th Street, Dallas Center, IA 50063 USA; 3 Department of Animal Genetics and Breeding, Faculty of Animal Science, Warsaw University of Life Sciences, Ciszewskiego 8, 02-786 Warsaw, Poland; 4 Nutribiogen, Witkowska 15/1, 61-039 Poznan, Poland

## Abstract

Avian influenza (AI) is a devastating poultry disease that currently can be controlled only by liquidation of affected flocks. In spite of typically very high mortality rates, a group of survivors was identified and genotyped on a 600K single nucleotide polymorphism (SNP) chip to identify genetic differences between survivors, and age- and genetics-matched controls from unaffected flocks. In a previous analysis of this dataset, a heritable component was identified and several regions that are associated with outcome of the infection were localized but none with a large effect. For complex traits that are determined by many genes, genomic prediction models using all SNPs across the genome simultaneously are expected to optimally exploit genomic information. In this study, we evaluated the diagnostic value of genomic estimated breeding values for predicting AI infection outcome within and across two highly pathogenic avian influenza viral strains and two genetic lines of layer chickens using receiver operating curves. We show that genomic prediction based on the 600K SNP chip has the potential to predict disease outcome especially within the same strain of virus (area under receiver operating curve above 0.7), but did not predict well across genetic varieties (area under receiver operating curve of 0.43).

## Background

Avian influenza (AI) is a devastating disease and the current approach that is used to control it is based on isolation and extermination of affected flocks in order to stop the spread of the virus. Alternative approaches, including the use of vaccines, have been only 60% effective in chickens [1, 2] due to the high mutation rate of the virus and lack of cross-protection between viral strains. Moreover, most countries do not allow importation of vaccinated birds due to the inability to distinguish between antibodies originating from the vaccine versus infection. Even in the case of high mortality rates caused by highly pathogenic avian influenza (HPAI) infections, typically above 85% in unvaccinated birds [3], there is usually a limited number of birds that survive following infection. Factors that underlie individual survival are likely multifactorial, but probably include a strong genetic component. Susceptibility to infectious disease (including influenza A virus) is highly heritable in humans [4–6] and there is evidence from inbred lines of chickens that this is also true for viral diseases in poultry, including AI [7–10]. A genetic approach using genotypes from the 600K Affymetrix single nucleotide polymorphism (SNP) chip was undertaken by Drobik-Czwarno et al. [11] to identify differences between survivors of two HPAI outbreaks (H5N2 in the US and H7N3 in Mexico) and their genetics- and age-matched controls from unaffected flocks. Heritability of survival to HPAI was estimated to be between 0.18 and 0.24, which indicated that almost 20% of the differences in survival could be attributed to genetics [11]. In addition, several of the genomic regions identified were associated with survival but none with a major effect, indicating a complex polygenic nature of resistance to the disease.

An alternative to searching for causal mutations, is to use high-density SNP genotypes for genetic improvement of disease resistance by using all genetic markers across the genome to predict breeding values of selection candidates through the concept of genomic prediction and selection [12]. Genomic prediction involves the estimation of the effects of all genetic markers on the phenotype in a training dataset, followed by the use of these estimates to predict the genetic or breeding value of selection candidates based only on their SNP genotypes across the genome. If there is a significant genetic component to resistance, this prediction and selection method can be effective even if no clear signals are obtained from genome-wide association studies (GWAS).

In clinical epidemiology, the receiver operating characteristic (ROC) curve is often used to evaluate diagnostic methods in terms of their ability to distinguish between healthy and sick subjects. This method has several advantages, including independence of prevalence of disease or choice of the decision criterion [13]. In this study, we used ROC curves to evaluate the diagnostic value of genomic breeding values for predicting AI infection outcome within and across different viral strains and genetic lines of layer chickens.

## Methods

Three sets of samples were used for this study (see [11] for a more detailed description): Hy-Line US commercial samples (205 survivors and 397 controls) from the 2015 H5N2 outbreak, Hy-Line Mexico commercial samples (480 survivors and 176 controls) from a 2012 H7N3 outbreak, and non-Hy-Line US commercial samples (47 survivors and 45 controls) from the same 2015 H5N2 outbreak in the US. All birds were from White Leghorn varieties. Blood samples from HPAI survivors and their age- and genetics-matched unaffected controls were collected on FTA Elute Microcards (GE Healthcare, Piscataway, NJ). Controls were selected from contemporary Midwest flocks that were not affected by HPAI because it was impossible to collect samples from dead birds on the affected farms for biosecurity and practical reasons. Because mortality in the H5N2 outbreak was higher than 99%, it was assumed that age-matched random birds from the same genetic varieties would be susceptible. DNA was extracted and genotyped on the 600K Affymetrix Axiom chicken SNP panel [14]. After quality control, 420,458 SNPs were retained for analysis, including some that were fixed within some subsets of samples. In case-control studies, population stratification is always a concern because it can lead to spurious associations. However, using multidimensional scaling analysis, Drobik-Czwarno et al. [11] showed that there were no structural differences between survivors and controls within the analysed populations.

The phenotype of survivor/control (0/1) was analysed using the BayesB method by fitting all SNPs simultaneously, in GenSel [15], separately for each dataset. The proportion of SNPs assumed to have no effect on survival was set to 0.999 in order to keep the number of SNPs in the model on the same scale as the number of available phenotypic observations. A mixed linear model with the fixed effect of an overall mean and random additive effects of SNPs was fitted. The length of the chain was 52,000 iterations with the first 2000 discarded as burn-in. Priors for variance components were 0.05 for genetic variance and 0.15 for residual variance, which is equivalent to a heritability of 0.25, with 4.2 degrees of freedom for residual variance to reflect the high level of uncertainty. Posterior estimates of SNP effects were used to compute genomic breeding values for survival to HPAI of genotyped birds that were not in the training data.

To verify the potential of genomic prediction for genetic improvement of AI resistance, random fivefold cross-validation was performed within Hy-Line US and Mexico commercial samples, as well as prediction of the Hy-Line US commercial samples from the Hy-Line Mexico commercial samples, to represent prediction across different virus strains, and prediction of non-Hy-Line US commercial samples from Hy-Line US commercial samples, to represent prediction across different genetic varieties of layer chickens. The usefulness of the genomic estimated breeding values (GEBV) for predicting disease outcome was summarized by ROC curves. Sensitivity (true positive rate) and specificity (true negative rate) were calculated across the whole range of thresholds to classify birds as survivors versus controls based on their GEBV, i.e., for a given GEBV threshold, sensitivity is the proportion of survivors among birds with GEBV above this threshold and specificity is the proportion of controls among birds with GEBV below the threshold. Sensitivity was plotted against (1-specifity) (= false positive rate) to create the ROC curves, together with a 45° line that represents random assignment of survivors and controls. Summary statistics of area under the curve (AUC) and significance test for AUC = 0.5 (or breeding values being equivalent in predictive ability to random assignment) were performed using the program easyROC [16].

## Results and discussion

Windows explaining more than 1% of the genetic variance within each dataset are included in Table 1. A 1-Mb window on chromosome 1 at 126 Mb consistently explained the largest amount of genetic variance in Hy-Line Mexico commercial samples (27 to 32%). This is in agreement with the strongest association signal identified by Drobik-Czwarno et al. [11] on chromosome 1, using different GWAS on the complete dataset. In the Hy-Line US commercial samples, the windows explaining the largest proportion of genetic variance differed between the folds (Table 1) and the estimates of variance explained were smaller than in the Hy-Line Mexico commercial samples. This suggests a more polygenic determination of survival of infection with the H7N3 virus than with H5N2. The use of birds from unaffected flocks as controls instead of dead birds from the respective affected flocks could have reduced power of the analysis and decreased the accuracy of genomic prediction. However, because mortality was very high (> 99%), the risk of including some potential survivors in the control group is expected to be limited.

**Table 1:** Location of the 1-Mb regions that explained more than 1% of genetic variance (%Var) for different datasets and the probability of that region having a nonzero effect (*P* > 0)

Data set	Chromosome	Position (Mb)	Number of SNPs	%Var	*P* > 0^b^
Mexico Hy-Line1^a^	1	126	306	32.1	1.0
Mexico Hy-Line2	1	126	306	28.1	1.0
Mexico Hy-Line2	29	0	5583	1.0	1.0
Mexico Hy-Line3	1	126	306	27.1	1.0
Mexico Hy-Line3	12	12	608	2.9	0.7
Mexico Hy-Line3	29	0	5583	1.2	1.0
Mexico Hy-Line4	1	126	306	28.8	1.0
Mexico Hy-Line4	4	69	340	1.4	0.6
Mexico Hy-Line5	1	126	306	26.0	1.0
US Hy-Line1	1	167	438	3.0	0.5
US Hy-Line1	20	3	615	1.1	0.5
US Hy-Line1	13	11	561	1.0	0.5
US Hy-Line1	1	166	352	1.0	0.4
US Hy-Line2	15	1	659	1.7	0.6
US Hy-Line2	15	0	422	1.1	0.5
US Hy-Line2	29	0	5583	1.0	1.0
US Hy-Line3	1	32	283	2.0	0.4
US Hy-Line3	15	1	659	1.9	0.6
US Hy-Line3	15	0	422	1.1	0.5
US Hy-Line4	1	71	364	1.0	0.4
US Hy-Line4	29	0	5583	1.0	1.0
US Hy-Line5	4	84	433	3.4	0.4
US Hy-Line5	1	167	438	2.3	0.5
US Hy-Line5	9	16	629	1.2	0.5
US Hy-Line All	7	28	497	3.0	0.4
US Hy-Line All	1	32	283	1.7	0.4
US Hy-Line All	9	16	629	1.1	0.5
US Hy-Line All	1	167	438	1.1	0.4

^a^The number at the end of the dataset name refers to the fold number of the fivefold cross-validation

^b^*P* > 0 was calculated as the proportion of MCMC iterations in which at least one SNP from that window was fitted in the model with nonzero effect

**Fig. 1:**
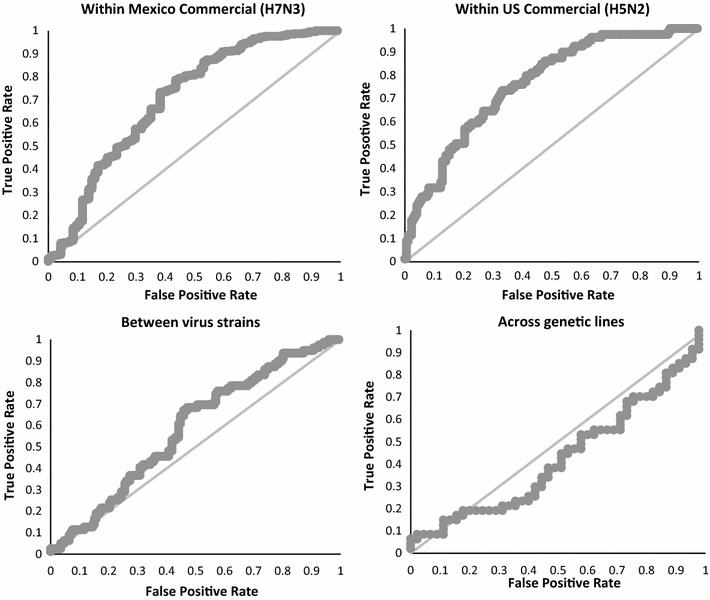
ROC curves for predicting AI resistance. Top left panel: within Mexico commercial (H7N3) data; top right panel: within US commercial (H5N2) data; bottom left panel: between virus strains; bottom right panel: across genetic lines

The ROC curves are in Fig. 1 and AUC results are in Table 2.

**Table 2:** Summary statistics of the area under the ROC curve including confidence interval and statistical test for differences from the value of 0.5 expected under random classifier

Training-validation scenario	Area under the ROC curve				z	*P* value
	Mean	SE	Minimum	Maximum		
Within US Hy-Line	0.76	0.03	0.70	0.82	8.41	4.0E−17
Within Mexico	0.71	0.03	0.64	0.77	6.20	5.7E−10
Across virus strains	0.58	0.04	0.51	0.66	2.25	0.02
Across genetics	0.43	0.06	0.31	0.55	− 1.14	0.25

The ROC curves show significant improvement (Table 2) in accuracy of prediction compared to random assignment for all tested scenarios, except when predicting across different genetic varieties of chickens. The best diagnostic performance of GEBV was observed when training and validation were within the same virus strain and the same genetic variety (Table 2). In spite of differences in the windows that explained the largest proportion of genetic variance between the different virus strains, prediction from Mexico to US Hy-Line samples was significantly better than random assignment (AUC = 0.58, *P* = 0.02). These results show that genomic predictions based on the 600K SNP chip have potential to predict disease outcome, especially within the same strain of virus, but do not predict well across genetic varieties. Methods to implement this type of crossbred performance for pure line selection using genomic data was discussed by Ibánẽz-Escriche et al. [17].

Poor performance of genomic prediction across layer lines for egg production was previously reported by Calus et al. [18] using genotypes from a 60K SNP chip and interpreted as evidence of low consistency of linkage disequilibrium between SNPs and QTL between chicken lines. This limitation is expected to be overcome by the use of high-density genomic data (up to the sequence level) but so far sequencing costs have been prohibitive for generating large-scale training populations based on sequence data in chickens and the gains in accuracy for within-line predictions using sequence data have been limited [19]. In addition, if the SNP chip density is higher, a larger number of samples is required to estimate genotype effects accurately, which with the extremely high mortality in HPAI outbreaks may make it very difficult to collect sufficient numbers of survivor samples. Thus, at this point, the use of sequence data appears more promising for the identification of causal mutations than a practical application for genomic prediction.

## Conclusion

Genomic predictions based on the 600K SNP chip have potential to predict avian influenza infection outcomes, especially within the same strain of virus but do not predict well across genetic varieties.
